# Molluscicidal Activity of Some *Solanum* Species Extracts against the Snail *Biomphalaria alexandrina*

**DOI:** 10.1155/2009/474360

**Published:** 2010-02-08

**Authors:** Gehad T. El-Sherbini, Rawia A. Zayed, Eman T. El-Sherbini

**Affiliations:** ^1^Department of Parasitology, Faculty of Pharmacy, October 6 University Cairo (Formerly Sinai University), 11471 El Arish, Egypt; ^2^Department of Zoology, El Nahda University, Beni Sweif, Egypt; ^3^Department of Pharmacognosy, Faculty of Pharmacy, Zagazig University, Zagazig, Egypt

## Abstract

*Background*. Snails' species are associated with transmission parasitic disease as intermediate host. Biological control stands to be a better alternative to the chemical controls aimed against snails. The search of herbal preparations that do not produce any adverse effects in the non-target organisms and are easily biodegradable remains a top research issue for scientists associated with alternative molluscicides control. *Method*. Solvent extracts of fresh mature leaves of *S. nigrum, S. villosum, and S. sinaicum* were tested against *Biomphalaria alexandrina*, a common intermediate host of *schistosoma mansoni*. A phytochemical analysis of chloroform: ethanol extract was performed to search for active toxic ingredient. The lethal concentration was determined. *Results*. Extracts isolated from mature leaves of *Solanum* species were found to be having molluscicidal properties. *S. nigrum* extract was recorded as the highest mortality rate. When the mortality of different solvent extracts was compared, the maximum (*P* < .05) mortality was recorded at a concentration of 90 ppm of ethanol extract of *S. nigrum*. *Conclusion*. Extract of mature leaves of *S. nigrum* exhibited molluscicidal activity followed by *S. sinaicum* and the less one was *S. villosum*. The study provides considerable scope in exploiting local indigenous resources for snails' molluscicidal agents.

## 1. Background

Schistosomiasis is a parasitic disease that affects 200 million people in different countries [1], and is frequently considered the second most important parasitic disease after malaria among the infectious diseases of tropical and subtropical countries [2], while being the third most prevalent parasitic disease in the world in terms of overall morbidity burden, socioeconomic and public health importance, and human impact [2]. Use of molluscicides to eradicate the snail vector is considered the method of choice to eliminate schistosomiasis [3]. Several different strategies have been used to control snail populations. Although praziquantel is available and generally very effective, the treatment is expensive and not always successful [3, 4]. In poor countries where schisotsomiasis is common, biological control of the snails that serve as intermediate hosts for Schistosoma and Fasciola, appears feasible and cost effective. Control of the intermediate host disrupts the life cycle of the parasite, stopping the transmission of infection. Synthetic organic molluscicides have been widely used for the effective control of vector snails [5]. However these molluscicides are considered toxic to nontarget animals and may have long-term detrimental effects on the aquatic environment [3, 6]. Medicinal plants represent the oldest and most wide spread form of medication known to man and have become the focus of attention as source of molluscicidal agents, since they are less expensive and less hazardous to the environment than their synthetic counterparts [3]. The high costs and toxicity of synthetic molluscicides have stimulated renewal interest in plant molluscicides [5, 6].

 The leaves of many species of Solanum have molluscicidal properties but relatively little work has been carried out on their possible application in field trials. Before field trials are started, more laboratory testing is, however, necessary to determine the (MLC50) minimum lethal concentration at 50 ppm, values for different species, and toxicity to nontarget organisms. Egyptian S. nigrum extracts were very effective at controlling intermediate host of parasites causing human schistosomiasis and fascioliasis [7]. In the present study 3 Solanum species (S. nigrum, S. siniacum, and S. villosum) were examined for molluscicidal properties. 

 Solanum villosum is a common weed known as red-fruit night shade and is Ayurvedic herb with multiple medicinal properties [8]. Solanum siniacum plants are isolated from the high lands of Tin (wilderness) desert of North Sinai, the mountains, known to be rich in plant diversity. The aim of this study was to screen the 3 Solanum species for molluscicidal activity, against the adult B. alexandrina snails, and compare relative toxicity of each plant species. B. alexandrina is the main snail host of Schistosoma mansoni, a human pathogen in Egypt.

## 2. Materials and Methods

### 2.1. Test Snails

Adult *B. alexandrina* (Shell diameter: 12–14 mm) was collected from irrigation canals in Giza and Dakahlia Governorates. Uninfected snails, that is, those that did not show patent trematode infections, were maintained in the laboratory conditions for seven days before being used in our molluscicidal tests. Ten snails were then allocated to each of the groups and immersed in either untreated dechlorinated tap water or aqueous extract treated dechlorinated water (positive & negative controls). Preparations of the plant extracts and toxicity test protocols were adapted from those described by Brackenbury and Appleton in [9, 10].

### 2.2. Plant Material

The plants examined in this study were selected on the basis of ethnopharmacological information indicating their medical uses in schistosomiasis control in the endemic areas. The plant species were collected locally from their natural habitat, *S. nigrum* from Nile region (Skarkia), *S. villosum*, and *S. sinaicum* were collected from Sinai proper during the period from March 2007 to February 2008, and identified by a plant taxonomist (Dr. Hesham El- Shamy, Faculty of Agriculture, Zagazig University, Egypt). A voucher specimen for this collection has been deposited in the herbarium of the Department of Horticulture, Faculty of Agriculture, Zagazig University, Egypt.

The molluuscicidal activity of the leaves extracts against the snails was assessed to determine the toxicity.

### 2.3. Preparation and Preservation of Plant Extracts

One kilogram (1 kg) each of the air-dried plant leaves were ground into fine particles and extracted twice with 2.5 litres each of alcohol at room temperature for 72 hours with shaking. The total extracts were filtered, then concentrated to dryness in vacuo under reduced pressure in a rotary evaporator at 40 + 5°C and finally yielding crude total extract of the plant leaves. Ethanol extract prepared by soaking leaves powder over night in cold 70% ethanol. 

### 2.4. Preparation of Plant Extracts in Different Solvent Systems

The different plant extracts were prepared using four solvents, namely petroleum ether, chloroform, acetone and methanol applying one after another. The extracts were collected separately. Each solvent extract was filtered, concentrated by evaporation in a rotary evaporator and the solid residues were weighed. The total yield of each extract from 1kg of leaves was: petroleum ether extract, 4.5 g, chloroform extract, 6.4 g, ethyl acetate extract, 4.00 g; and methanol extract 5.6 g. Different test dilute solutions, ranging from 10 to 1000 mg/L, (i.e, ppm) were prepared from the stock solutions, using deionized and dechlorinated water, to determine the LD_50_ and LD_90_ values.

### 2.5. Molluscicidal Activity Tests

Molluscicidal evaluation of the plant extracts was performed according to WHO guidelines [11]. Groups of 10 uninfected snails were placed in glass tanks (containers) with some sand, snail food, and 1000 mL of deionized and dechlorinated pond or tap water bubbled with atmospheric air. Tests were carried out at room temperature (26 + 1°C). In each setup, the snails were prevented from crawling out of the glass container by means of a fine stainless steel mesh placed above the water surface. The test snails were challenged with various concentrations of the plant extracts (ranging from 10 to 1000 mg/L (ppm). After 24 hours of exposure to the plant extracts, the snails were transferred to fresh dechlorinated and deionized water and maintained there for another 24 hours. Death of the snails was determined and confirmed by the absence of heartbeat and lack of reaction to irritation of the foot with a blunt wooden probe to elicit typical withdrawal movements. Control solutions were also made with deionized and dechlorinated tap water. Control experiments were performed with deionized and dechlorinated water alone (negative control) or with niclosamide (Baylucide WP70) (positive control). Molluscicidal test with each plant extract dose was separately repeated three times. The snails were neither fed nor disturbed during the exposure and recovery periods. LD_50_ and LD_90_ (referring to the plant extract doses in ppm that kill 50% and 90% of the test snails, respectively) were determined by the methods of Leitchfield and Wilcoxon (1949) [12] with 95% confidence limit. Plant extracts that cause, no mortality at 1000 ppm were considered inactive and were not investigated further.

## 3. Results and Discussion

### 3.1. Bench Side Observation

Each snail in the untreated water tanks (controls) initially withdrew into its shell but resumed normal activity after about 45 minutes, moving around the container with its foot extended. When a mechanical stimulus was applied to the footsole, the snail immediately retracted into its shell. In the test snails, the toxic effects of the active plant extracts become evident. There was either a partial retraction (withdrawal response) in the partially dead snails or no retraction at all (in the dead snails) to mechanical stimulation of the foot-sole with a blunt needle. There was a visible swelling of the cephalopodal mass. Development of hemorrhagic “blisters” over the ventral surface of the foot-sole was also noted. High doses of the active plant extracts caused the cephalopodal mass of each snail to become severely swollen, turgid and failing to respond to mechanical stimulation with a blunt needle. Mucous secretion was observed over most of the foot.

### 3.2. Experimental Findings

**Figure 1:**
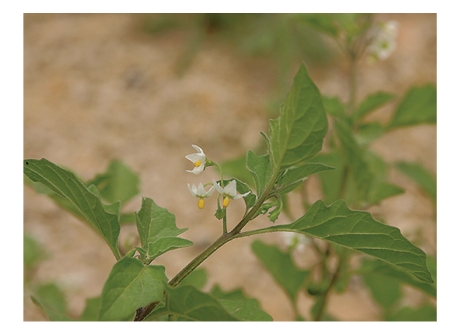
*S. nigrum*.

**Figure 2:**
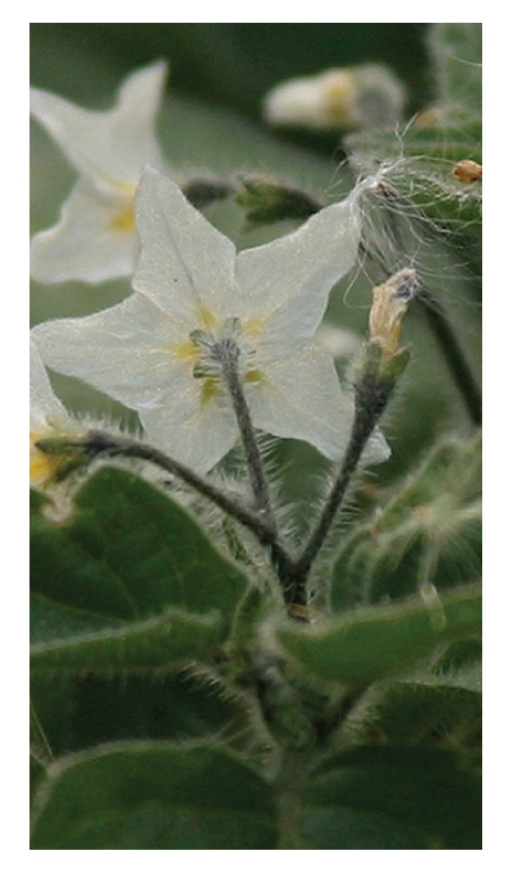
*S. villosum*.

**Figure 3:**
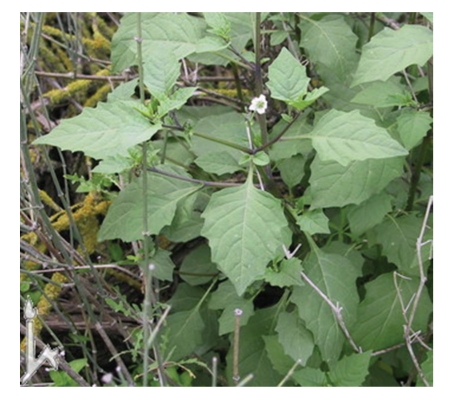
*S. sinaicum*.

From Tables 1 and 2 The LC_50_, and LC_90 _for leaf extracts of *S. nigrum, S. sinaicum*, and S. *villosum* are provided. The present data revealed that ethanol extract of *S. nigrum* leaves showed the highest molluscicidal activity (LC_90_ = 5.95 mg/L), followed by *S. sinaicum* (6.04 mg/L) and *S. villosum* (8.95 mg/L). These similar to other reported data [13]. The richness of the flora in parts of the world where snail transmitted diseases is endemic. Probably suggests that many plants with molluscicidal properties remain to be discovered. Several promising plant molluscicides have been identified. Previous studies have shown that potency levels of plant samples vary significantly according to season and geographical location of the plants, such unpredictable trends in the potency of plant molluscicides militate, against their selection of control program [14]. Over 100 species of *Solanum *are indigenous to Africa and several of these species have been developed there [15]. Several *Solanum* species of the family Solanaceae are widely used as leafy vegetables as source of fruits and medicine in different countries, In this study*, S. nigrum, *(Figure 1),* S. villosum,* (Figure 2) and *S. sinaicum* (Figure 3), which in its features like *S. nigrum* were tested against *B. alexandrina* snail, the intermediate host of *Schistosma mansoni*. The present investigation showed the effect of exposure of the different concentration of the three species of* Solanum* against the snails, and the results were agreed with some other results recorded by [7, 8, 16, 17]. 

After exposure to the active plant extracts examined in the present study, the snails showed several behavioural responses, including the “distress syndrome" described for other planorbid species by [18–21] indicative of intoxication. Swelling of the tissues was not restricted to the tentacles, but involved the whole cephalopodal mass. According to [21], the inference from this observation is that the tissue of the cephalopodal mass had accumulated water, which caused haemorrhage at lethal concentration of the active plant extracts. Nevertheless the observation made in this study suggest that the toxic principles in the active plant extracts disturbed the permeability of the foot-sole surface epithelium by preventing its normal osmo-regulatory function [21]. The toxic effect of the sublethal doses of the plant extracts were, however, reversible after exposure if the snails were moved to toxic extract-free water for a recovery period. This observation is in agreement with the findings of Herry and Aldrich (1963) [18], and Van Aardi and Coertze (1981) [20] for *Bulinus tropicus* and *Biomphalaria *after exposure to copper.

## 4. Conclusion

The results of the presented study have shown that *S. nigrum, S. sinaicum, and S. villosum *possess molluscicial properties against the snail *B. alexandrina*. Ethanol extracts of of *S. nigrum* had a stronger molluscicidal activity than the other extract, and therefore it is the most suitable for biological application which offers a potentially simple, readily available, inexpensive and environmentally safe molluscicidal agent of plant origin for controlling human schistosomiasis. Use of plant molluscicides not only may eliminate the economic burden of importing expensive synthetic molluscicides, but also could stimulate growth of small-scale industries in developing countries. If plant molluscicides are to be applied successfully and in longterm they will sustainable control of schistosomiasis.

##  Authors' Contributions 

The first author conceived the idea, designed the experiments, trained assistants for sample collection, interpreted the experimental results, and provided critical revision of the manuscript. 

 The Second author carried out the laboratory bioassay experimentation and phytochemical analysis of the extract. 

 The third author carried out the data analysis and assisted in the planning of the study.

All authors contributed to manuscript preparation and approved the final manuscript.

## Figures and Tables

**Table 1:** The results of molluscicidal evaluation of the 3 plants examined.

Plants	LD_90_ values for the snail used
(1) *Solanum nigrum *	100–200 ppm
(2) *Solanum sinaicum *	100–200 ppm
(3) *Solanum villosum *	200–400 PPm
(4) Positive control Niclosamide (Bayluscide)	0.20–0.8 ppm

Key: 0.1–10 ppm = very strong molluscicidal activity. 50–100 ppm = Moderate to strong. 100–200 ppm= Mild to moderate. 200–400 ppm = Weak to mild. Niclosamide (Bayluscide), used as (positive control) reference molluscicide, killed all the snails at a dose of 1 ppm. On the contrary, none of the snails (in the negative control) treated with deionized, dechlorinated water alone died.

**Table 2:** LC50 and LC90 for solvent extracts of leaves of *S. nigrum, S. villosum*, and *S. sinaicum* against *B. alexandrina* after 24 hours at room temperature.

	Activity (mg/liter) 24 hours					
Type of solvent	*S. nigrum*		*S. Villosum*		*S. sinaicum*	
	LC_50_	LC_90_	LC_50_	LC_90_	LC_50_	LC_90_
Pet. Ether	4.2	8.62	6.33	11.02	5.8	9.94
Chloroform	70.75	142.7	90.0	175.7	82.5	164.7
Acetone	6.55	11.7	9.67	12.9	8.7	11.2
Methanol	6.9	16.2	8.4	18.3	7.8	17.5
Ethanol	2.98	5.95	4.88	8.95	3.19	6.04
